# Constructing osteoarthritis through discourse – a qualitative analysis of six patient information leaflets on osteoarthritis

**DOI:** 10.1186/1471-2474-8-34

**Published:** 2007-04-11

**Authors:** Janet C Grime, Bie Nio Ong

**Affiliations:** 1 Primary Care Musculoskeletal Research Centre, Keele University, Keele, ST5 5BG, UK

## Abstract

**Background:**

Health service policy in the United Kingdom emphasises the importance of self-care by patients with chronic conditions. Written information for patients about their condition is seen as an important aid to help patients look after themselves. From a discourse analysis perspective written texts such as patient information leaflets do not simply describe the reality of a medical condition and its management but by drawing on some sorts of knowledge and evidence rather than others help construct the reality of that condition. This study explored patient information leaflets on osteoarthritis (OA) to see how OA was constructed and to consider the implications for self-care.

**Methods:**

Systematic and repeated readings of six patient information leaflets on osteoarthritis to look for similarities and differences across leaflets, contradictions within leaflets and the resources called on to make claims about the nature of OA and its management.

**Results:**

Biomedical discourse of OA as a joint disease dominated. Only one leaflet included an illness discourse albeit limited, and was also the only one to feature patient experiences of living with OA. The leaflets had different views on the causes of OA including the role of lifestyle and ageing. Most emphasised patient responsibility for preventing the progression of OA. Advice about changing behaviour such as diet and exercise was not grounded in lived experience. There were inconsistent messages about using painkillers, exercise and the need to involve professionals when making changes to lifestyle.

**Conclusion:**

The nature of the discourse impacted on how OA and the respective roles of patients and professionals were depicted. Limited discourse on illness meant that the complexity of living with OA and its consequences was underestimated. Written information needs to shift from joint biology to helping patients live with osteoarthritis. Written information should incorporate patient experience and value it alongside biomedical knowledge.

## 1. Background

In current NHS policy, the provision of comprehensive information for patients is viewed as a necessary resource for effective self management of health [1-3]. Leaflets for patients are one form of information, though many patients receive neither written nor spoken information [4]. What understanding patients get about their health problem from written information will depend not only on the facts that are included but also the discourse employed [5]. Silverman argues that texts should not be treated as if they were correct or incorrect written representations of reality but rather as accounts which help to construct the reality [6], and Potter and Wetherell (1987) suggest there is a great variation between accounts because they have different tasks to perform [7]. Thus the language of the patient information leaflet is not simply an intermediary which enables an understanding of the 'truth' about health and disease but actually helps construct particular understandings of health and disease.

This can be seen in a study by Coulter and colleagues which evaluated written patient information [8]. They found a common failing of leaflets was to give an over optimistic view of treatment emphasising the benefits while downplaying the adverse effects, and to hide medical uncertainty. When reviewing one leaflet on back pain some patients liked its positive approach but others disliked its patronising and victim blaming style.

*A woman who had been suffering from chronic back pain for some time felt the leaflet judged her negatively and implied (with statements like 'back pain need not cripple you unless you let it') that her inability to do certain things was her own fault.' *(Page 71 [8])

The woman interpreted the information to mean that she had control over whether or not chronic back pain resulted in disability. The particular phrase to which she took exception drew on a biomedical rather than a social model of disability [9]. In a social model the role of external factors in the socioeconomic environment, and over which individuals may have little control, is seen as crucial in the determination of whether impairments such as back pain are disabling.

Dixon-Woods (2001) identified two distinct discourses in patient information materials [10]. One (patient education) stems from a biomedical perspective and is concerned with educating patients in order primarily to bring their thinking in line with health professionals and increase compliance with treatment. The other discourse (patient empowerment) values patient agendas and is concerned with empowering patients and engendering a more equal relationship between patients and professionals. Dixon Woods points out that patient and professional interests often coincide and it is too simplistic to see the two discourses as oppositional. However they differ in their orientation to the patient. A patient education discourse essentially sees patients as passive and uninformed whereas a patient empowerment discourse conceptualises patients as competent and resourceful.

Most condition based, written information for patients is produced by health professionals, and uses a patient education discourse to address aspects of the disease[11]. In contrast NHS policy concerning chronic disease self-management recognizes the expertise of patients[12].

*An observation often made by doctors, nurses and other health professionals who undertake long-term follow-up and care of people with particular chronic diseases like diabetes mellitus, arthritis or epilepsy is "my patient understands their disease better than I do." This knowledge and experience held by the patient has for too long been an untapped resource*. (The expert patient: a new approach to chronic disease management for the 21st century 2001 DH)

In the Expert Patients Programme patients with long term conditions are seen as being knowledgeable about their condition. Skelton contrasts two models of patient education [13]. One is biomedically centered and tends to work with an assumption that patients will change their behaviour simply by being given information. This resonates with a patient education discourse. The other is patient centered education and in line with a patient empowerment discourse considers that influencing individual illness behaviour is complex, and has to take into account the broader context of a patient's life experiences. Despite research critiquing the paternalism of a patient education discourse, and policy promoting a patient centered approach, the biomedical model of patient education continues to dominate [14].

Osteoarthritis is the most prevalent form of arthritis and the most common reason for loss of mobility in those aged 65 and over [15,16]. People with OA are amongst the patient groups the government wishes to see more involved in their own care. The purpose of this study was to compare and contrast the discourse in information written for patients with OA from a variety of sources, in order to see how OA was constructed and to consider the potential implications for self-care.

## 2. Methods

Six leaflets were selected on the basis that they were easily available either over the Internet, through patient organisations or places that people with OA might visit such as pharmacies. They came from three different types of source, health care providers (UK National Health Service – NHS Direct, and British United Provident Association – BUPA), charitable and voluntary organisations (Arthritis Care – AC, and Arthritis Research Campaign – **arc**) and the medical profession (British Medical Association – BMA, and a doctor led website – Patient UK) The BMA booklet included arthritic conditions other than osteoarthritis. So in the BMA booklet analysis was restricted to the chapter on osteoarthritis and the generic chapters for arthritis and rheumatism on treatments and living with the conditions. (See Table 1)

The leaflets were analysed from a discourse analysis viewpoint in which text is not viewed simply as words that are written down but as a form of social practice [17]. Discourse analysis is concerned with how certain types of knowledge and power have taken precedent over others in producing the text and considers that texts gain authority from building on more powerful resources [18].

The texts were read repeatedly to look for common categories. From this initial coding three main information themes were identified;

• OA as joint pathology

• the causation of OA and implications for lifestyle and growing older in those with OA

• the management of OA

Each leaflet was systematically compared with the others in relation to the three themes to look for similarities and differences across leaflets, contradictions within leaflets and the resources that were called on to make claims about the nature of OA and its management. The significance for self care practice was considered by relating the findings to the literature on lay experiences of living with arthritis.

Before the findings are described the concepts of disease and illness we have used will be defined. In the paradigm of modern scientific medicine a disease is a disturbance of normal biological functioning, which can be detected by a scientific test. However symptoms may be experienced for which no pathological lesion can be found and vice versa. In the 1970's Eisenberg, an anthropologist, contrasted the patients' concern with the experience of symptoms and their effect on social functioning i.e. illness, with the doctors' focus on symptoms as indicators of underlying pathology in the body i.e. disease [19].

## 3. Results

The six leaflets will be compared and contrasted in relation to their three main areas of discourse identified from the thematic analysis.

### 3.1 Discourse of OA as a disease and as an illness

The biomedical discourse of OA as a disease, bringing together biology, epidemiology, biomechanics, symptoms and medical treatment, was predominant in all six publications. The focus was on joints and how they work. Except for NHS Direct all the leaflets had drawings of joints in cross section showing the loss of cartilage which characterises osteoarthritis as a disease.

However four leaflets (NHS Direct, **arc**, Patient UK and BMA) stated that some people with evidence of thinning of the cartilage are asymptomatic.

*Many people have no symptoms at all and find out they have osteoarthritis only when an X-ray is taken for some other reason*. (BMA)

A different set of leaflets (**arc**, Patient UK and AC) made a related point about the poor correspondence between the degree of pathology seen on an X-ray and the severity of symptoms. Thus the biological changes to the joint defined the disease but not the experience of symptoms – the illness. (See definition at end of methods section.) There was no discussion about the two different ways of seeing OA – as a disease or an illness. When the leaflets talked about OA it was not always clear whether they were referring to the disease or the illness, or where they were clear a biomedical disease discourse was privileged.

*Although the weather may temporarily affect the symptoms, it does not affect the actual arthritis itself*. (AC)

'Actual' arthritis is taken to mean what is happening to joint pathology not the symptoms that the patient experiences. The AC leaflet also implied that people with evidence of pathology could have symptoms which were imperceptible.

*Pain can vary in severity and can be so mild that many people don't even notice it*. (AC)

Postulating the existence of symptoms, which a patient may not experience, maintained the integrity of the disease discourse i.e. the existence of a relationship between pathology and symptoms. From an illness perspective the assertion makes less sense; if a pain is so mild to be imperceptible is it a pain?

Two leaflets did not move beyond biomedical aspects of OA as a disease (BUPA and NHS Direct). The BMA booklet had a chapter called 'Living with arthritis and rheumatism'. The focus of this though was to educate patients about exercise, joint protection and pain control, not to consider the broader issues of daily life with arthritis. **arc **did address psychological aspects of coping with OA and advised on the need to have a positive outlook

*Make every effort to make life fuller and more interesting than before. Your morale will drop after too much rest and inactivity, whereas hobbies and interests take your mind off your problems*. (**arc**)

The advice was not set in the context of people's lives and what making life fuller and more interesting might mean. A pen picture accompanying the advice shows an older woman sitting at a table with books, pen and paper. The leaflet had three case histories which were fictional, though the experiences of how the three people managed their OA **arc **described as typical. The case histories were biomedical rather than biographical and suggested that with a positive outlook, common sense and adherence to medical advice the impact of OA on daily life could be minimised. In contrast throughout the AC leaflet there were brief quotes from people actually living with OA.

*My knee and spine are more painful when the weather turns bad*.

*If I go out socialising, I accept I might feel a bit off colour the next day*. (Quotes from people with OA in AC leaflet)

The quotes illuminated the difficulties and trade offs that patients faced in real life. This was the only leaflet to use lay knowledge as a resource. However, the quote above about the effect of the weather on symptoms came soon after a heading entitled 'Myths'. The juxtaposition of myths and patient quote had the effect of suggesting that the patient's perception was a myth. Patients lived experience of arthritis was not well integrated into the text and the result was that lay knowledge was distanced from professional knowledge rather than complementing it.

The AC leaflet was also the only one to have a section concerned with caring for oneself as opposed to caring for one's joints, in which the individual nature of both illness and people's emotional response to illness was discussed.

*From time to time, your arthritis will get on top of you. Anger, frustration, uncertainty, depression and fear are all very understandable and very common*. (AC)

Compare this recognition of the individuality of those with arthritis with generic advice in the **arc **leaflet.

*Although osteoarthritis is often painful and upsetting, it usually does not cause crippling arthritis or severe deformity of joints. For most patients it will be more of a nuisance than a major problem*. (**arc**)

In **arc **the patient was viewed as one of a population of people with osteoarthritis; an average patient whose health could be categorised according to the degree of pathology in their joints. The distinction between problem and nuisance was a biomedical interpretation.

### 3.2 Discourse on causality, lifestyle and ageing in OA

#### 3.2.1 Causes of OA and lifestyle advice

A range of factors, which included age, genetics, gender, ethnic origin, joint injury, and over use of joints, were variously mentioned as being associated with the likelihood of developing OA. While acknowledging that the causes of OA were not known, NHS Direct, **arc **and AC wrote about causal relationships between risk factors and OA.

*For many people this *(obesity) *is an important factor in causing osteoarthritis especially at the knee*. (**arc**)

Lifestyle advice featured in all six leaflets. Body weight and exercise dominated. All agreed that losing 'excess' weight and regular exercise could help prevent worsening and/or relieve OA by reducing stress on the joints and strengthening the muscles around them. The importance of exercise was particularly emphasised in the BMA and AC leaflets. The BMA adapted political rhetoric to make the point.

*Exercise, exercise and exercise are probably the three most important factors in keeping the joints healthy! *(BMA)

All apart from BUPA were concerned with promoting exercise to help improve not only joint health but also general health. However, the BMA and the AC leaflet introduced the idea of a right kind and a wrong kind of exercise for OA and **arc **wrote of physiotherapists advising on correct exercises. Thus exercise was only helpful for OA if it was the 'right sort'. Advice on how much exercise to take resulted in more contradictory messages. While it can be seen from the quote above that the BMA was very keen to promote exercise, they also advised the patient to respect pain and not to push to continue with activities that made pain worse. AC and **arc **stressed the need to achieve an optimal balance between exercise and rest, though **arc **said that extra pain caused by exercise would be unlikely to damage a joint. Thus the reader was simultaneously advised to look after their joints by exercise and not to be afraid of moving them, but then to avoid straining them by overdoing things and to respect pain. The state of medical knowledge in relation to exercise and OA appeared uncertain, but there was no reference to this.

Most information was about lifestyle goals rather than advice on the process of their achievement. Nevertheless, the way that the goals were described indicated the authors' perception of the ease or difficulty of the process of attainment. **arc **and Patient UK encouragingly told the reader that even losing a few pounds would help. NHS Direct wrote of the need to 'shed excess weight' which made losing weight sound as straightforward as taking off an overcoat. AC advised 'sticking to your ideal weight' even though a patient quote in their leaflet showed that knowing about ideal weight was one thing and achieving it quite another.

*I know it's best not to be overweight with OA, but it's difficult*. (Quote from patient with OA in AC leaflet.)

The BMA booklet had a chart for readers to calculate their body mass index but the booklet said little about how to reach an ideal BMI.

In most of the information on lifestyle individualism was evident, where lifestyle was taken to mean those behaviours which have been identified as risk factors for disease and with an underlying assumption that individuals are able to choose a 'healthy' lifestyle by modifying their behaviour. The BMA saw modern lifestyles as a major reason why the prevalence of musculoskeletal problems had increased.

*The result of body abuse – lack of exercise, overweight, poor posture and overuse syndromes are the scourges of modern life in affluent societies. Abolishing these scourges would reduce not only locomotor problems but also diabetes, high blood pressure and heart disease*. (BMA)

Thus in some leaflets patients were held partially responsible for having developed OA. Ageing was handled in a rather different way from lifestyle and it is to this that we now turn.

#### 3.2.2 Ageing and OA

While all leaflets agreed that the occurrence of pathological changes of OA increased with age, except for Patient UK and BUPA they resisted the idea that OA was part of the natural ageing process. AC expressed this strongly.

*They (medical experts) no longer see osteoarthritis as being an inevitable part of ageing or a wear and tear disease, but more a challenge to fight*. (AC)

AC opposed the use of the term wear and tear since it suggested that OA was an inevitable consequence of growing older. However, a statement made a little later from the one above said:

*It is not known exactly why older people tend to develop it, but it is probably due to bodily changes which come with old age, such as the muscles becoming weaker, putting on weight and the body becoming less able to heal itself*. (AC)

In this explanation OA sounded as if it was the result of normal ageing processes and perhaps even wear and tear. The BMA booklet also found it hard to consistently disentangle what is and is not normal in old age. To begin with it said the idea that the *disease OA *is natural and inevitable in later life was too simplistic but soon after, that it was the idea that *disability *caused by OA is inevitable in later life, which was out of date. Severing the link between OA and normal ageing enabled OA to be viewed as a disease which should be a subject of biomedical research and which was a legitimate concern of doctors, but also one for which patients should take responsibility.

### 3.3 OA management discourse

#### 3.3.1 Role of health care professionals

Responsibility for preventing the onset and progression of OA was placed to some degree with patients.

*So to a certain extent the person with osteoarthritis is in control of his or her own outcome*. (**arc**)

*Most people can lead a full active life with osteoarthritis by properly managing the condition and making small, common sense alterations to life*. (AC)

While emphasising the importance of self-help there were differences between the leaflets in the extent to which patients should act autonomously. On nine occasions AC advised the patient to see their doctor before taking up exercise, changing their diet or trying alternative medicine. BUPA and NHS Direct did this once.

*Exercises must be prescribed by your doctor and done under medical or paramedical supervision. Physiotherapists are trained in this work*. (NHS Direct)

**arc **takes a more indirect approach pointing out that therapists can offer help and guidance if it is needed but left it to the readers' discretion. Likewise the BMA did not advise seeing the doctor before trying out exercises or changing diet but did consider that seeing a physiotherapist could be useful. While AC was keen that patients see their GP before embarking on specific self-help measures it was the only leaflet to use the word self-management and to promote self-management courses.

All leaflets anticipated that patients while helping themselves would have some contact with primary health care professionals in particular their GP. The amount of contact envisaged varied. The AC leaflet said it was important to visit the GP and that the patient and GP must work together to manage the patient's arthritis. While **arc **thought that patients would only need occasional advice from doctors and therapists.

BUPA placed least emphasis on self-help. Their leaflet was more concerned with the potential of medical intervention. More than a quarter of the leaflet was taken up with medicines and surgery. Surgery was presented as a fairly routine treatment whereas in the other leaflets it was shown as an option of last resort and reserved for the most severe cases. The orientation of the leaflets towards the use of drugs varied. BUPA was most and **arc **least enthusiastic. **arc **and Patient UK had different advice about the role of painkillers.

*(Painkillers) do not affect the arthritis itself but take the edge off pain and stiffness. They are best used occasionally for bad spells, or when extra exercise is likely*. (**arc**)

*It is best to take (paracetamol) regularly to keep pain away, rather than 'now and again' when pain flares up*. (Patient UK)

**arc**'s advice directly contradicted that in Patient UK. Neither gave the evidence for why analgesics should or should not be used regularly or indicated whether this is an area of choice for patients.

Most leaflets mainly conveyed the idea that for the majority of patients with OA, i.e. those with a less severe pathology who do not warrant surgery, biomedical treatments were limited to consulting health professionals for lifestyle advice and the use of painkillers. Nevertheless **arc **and BMA spoke reassuringly about the potential of biomedicine.

*Modern medicine has a lot to offer *(BMA)

#### 3.3.2 Non orthodox treatments

Unlike their discourse on orthodox treatments, BMA, **arc **and BUPA wrote with a degree of scepticism about complementary treatments.

*There are many complementary and alternative approaches to treating osteoarthritis, although the evidence that they work is usually only anecdotal*. (BUPA)

*A few (complementary medicines) like acupuncture, have a proven short-term pain-relieving effect. But many do no more than produce a 'placebo' effect (as when someone receiving a simple sugar pill actually believes it is making them better*) (**arc**)

Patient UK and NHS Direct did not include information on complementary therapies. The AC leaflet had conflicting ideas. On the one hand it made the point that complementary therapies could have a positive effect on symptoms and outlook on life but not the underlying condition. On the other hand it said that the Alexander technique can alleviate conditions caused by poor posture and that osteopathy can allow the body to heal itself. AC expressed some concern about the relationship between orthodox and complementary medicine.

*Complementary therapies can generally be used alongside orthodox treatment, although doctors may vary in their attitudes to them*. (AC)

The differences in the handling of orthodox and complementary therapies could be quite subtle yet succeed in raising suspicion about the latter. The BMA had boxes with guidelines for using simple analgesics, non-steroidal inflammatory drugs, long term steroids and complementary medicines. While the question of undesirable interactions between conventional and complementary treatments was flagged, such interaction between over the counter and prescribed medicines was not. The complementary medicine taker was also singled out for being told to relate the cost of treatment to effectiveness and recommended to keep a diary to monitor symptoms to assess if the complementary therapy had any effect.

All leaflets mentioned the food supplements glucosamine and chondroitin. In Patient UK, **arc **and NHS Direct they were incorporated into the biomedical discourse on treatment. AC and BUPA placed them in a separate section from medicines and the BMA put them within the treatment chapter but in their own section. Where food supplements were described alongside mainstream treatments they appeared to attract greater scientific certainty.

## 4.1 Discussion

Although the biomedical model dominated, there were variations as to how OA was seen and understood. In the BUPA leaflet OA was a natural part of the ageing process which biomedicine could successfully treat by means of drugs or surgery. In contrast information from AC, **arc**, BMA, and NHS Direct placed greater responsibility for preventing OA from starting and progressing with the patient. Patient UK's leaflet largely reported the facts of OA as understood from the results of medical research. Patient UK said that OA could be considered a normal part of ageing but that it was not always progressive. It did not make patient behaviour central to progression and presented a range of suggestions to help with symptoms, many of which did not require access to a doctor. **arc **portrayed OA as a not serious medical condition which scientific research would cure in time. Arthritis Care's leaflet was the only one to include an illness discourse albeit limited and not fully integrated. It was the only one to refer to self-management but at the same time repeatedly encouraged patients to get medical approval before embarking on any course of action that could be loosely considered a treatment.

The differences between the leaflets could be confusing if a patient were to try and follow the advice in all of them. However it was what they had in common – the dominance of the disease discourse – that arguably would potentially have more impact on developing people's abilities to self care [10]. In the paradigm of scientific medicine the human body is seen to be a tangible object that can be described and understood outside of the context it inhabits [20]. In the biomedical model the disease of osteoarthritis is viewed as a set of symptoms relating to underlying pathology, which is understood independently of the person with the symptoms. Illness on the other hand is the lived experience of symptoms including the consequences of their occurrence and the response of the ill person's wider social network [21]. One reason for a mismatch between the doctor's and patient's agenda in the consultation is that the doctor is often focusing on the disease and the patient on the illness [14]. This mismatch was perpetuated in five of the six leaflets reviewed; they were leaflets mainly about bodies rather than people [22].

Advice about how to self-care paid great attention to the biological effects of OA but much less to the social ones. OA is more likely to be found amongst the elderly. How those with OA understand the significance of symptoms is influenced by how they interpret normal ageing [23]. Sanders et al (2002) found that although the older people they interviewed experienced significant disruption from OA they tended to play down symptoms because disability was seen as a normal part of ageing and being independent valued as a sign of successful ageing [24]. Four leaflets separated OA from ageing, based on the fact that not all older people develop OA. However, it was not a convincing explanation since they described other musculoskeletal changes underlying OA which were part of the ageing process. There appeared to be a contradiction between the discourse of the bio-scientist based on epidemiological studies showing ageing to be a risk factor in OA, and the discourse of the NHS therapist wanting to encourage patient responsibility for preventing OA, which necessitated establishing that OA was a preventable condition.

Bury (1988) suggests a reorientation of the focus for care from repairing damage caused by disease to education and understanding for living with chronic illness [25]. Research has shown that many factors influence the kind of everyday self care decisions patients make [26] and that self care practices are learned over time [27]. The challenge is how to integrate expert advice with ways of managing chronic illness that people have developed for themselves. This poses a difficulty for written information which cannot be responsive to an individual situation. As a starting point though written information could include a patient perspective. For example studies have shown people's general resistance towards the long term use of medicines [28] and that many patients take the minimum dose of painkillers as a last resort [29,30]. Using painkillers in this way could be viewed as suboptimal pain relief stemming from patient ignorance [30] or a resourceful self care decision [29]. Advice on painkillers adopting the former stance is a biomedically centred model of education and the latter a patient centred one [13].

Advice in the six leaflets about changing behaviour such as diet and exercise was not grounded in lived experience. Advice was sometimes simplistic – 'shed weight', or assumed compliance with medical direction, and laid responsibility for change solely with the individual. There was no acknowledgement of psychosocial barriers to making lifestyle changes or indeed getting treatment [31] and little recognition of social determinants of health. Information about what a patient might expect to happen in a consultation for OA also appeared idealised. Sanders et al (2004) report patients' negative experiences of consultations in primary care with some GPs reinforcing a model of OA as a normal part of ageing and for which nothing could be done [31]. This is rather at odds both with the discourse in BMA and **arc **about the success of biomedicine, as well as the close ongoing relationship envisaged by AC between doctor and patient. AC's advice to check with the doctor did not recognise the reality that many patients are reluctant to consult unless they consider they have a legitimate reason to do so [32]. Checking information about the suitability of exercise regimes or stopping taking painkillers may not be considered a sufficient reason.

A consequence of the leaflets' focus on disease meant that the nature and meaning of the illness, together with patient experience of chronic symptoms and disability was by and large not addressed or used as a resource. The gap between lay and professional understanding of illness can be a common source of patient dissatisfaction in the consultation [14]. A professional response to a mismatch between lay and professional views is to want to educate patients [30] rather than understand the personal and cultural meanings of patients' experiences. If leaflets are to help patients in self care of a chronic condition like OA then they need to offer more than a biomedical narrative [33] and include evidence which draws on patients' experiences of living with arthritis.

## 4.2 Conclusion

A disease discourse dominated the leaflets. This impacted on how the causality and treatment of OA and the respective roles of patients and professionals were seen and understood. The limited discourse on illness meant that the complexity of living with OA and its consequences was underestimated. If written information is to help support self care then the focus needs to shift from joint biology to helping patients live with osteoarthritis. Written information should provide an illness discourse by incorporating and valuing patients' experience of OA.

## Competing interests

The author(s) declare that they have no competing interests.

## Authors' contributions

JG conceived and carried out the study, and drafted the manuscript. BNO contributed to the analysis and interpretation of data, and critically revised the manuscript. Both authors read and approved the final manuscript.

## Pre-publication history

The pre-publication history for this paper can be accessed here:



## Figures and Tables

**Table 1:** Profile of patient information leaflets on osteoarthritis (OA)

**Source**	**Arthritis Research Campaign arc**	**Arthritis Care AC**	**British Medical Association BMA**	**BUPA**	**NHS Direct**	**Patient UK**
**Organisation**	Charity Promotes medical research and education	Voluntary organisation working with/for people with arthritis	Doctors' professional body who produce booklets on a range of medical topics	Private health care provider	Part of NHS	Partnership between 2 doctors and EMIS a company which supports general practice electronic systems
**Publication title**	Osteoarthritis	Living with osteoarthritis	Understanding Arthritis and Rheumatism	Osteoarthritis	Osteoarthritis	Osteoarthritis
**Date of publication**	2004	2005	2005	No date but fact sheets are updated every two years	2003	2005
**Accessed**	Internet but also available from arc	Internet but also available from AC	Purchased from pharmacy	Internet but also available from BUPA	Internet	Internet
**Number of pages**	26	34	OA – 5 Generic Treatment – 15 Living with – 18	4	10	6
